# Why we still need drugs for COVID‐19 and can't just rely on vaccines

**DOI:** 10.1111/resp.14199

**Published:** 2021-12-30

**Authors:** Bruce W. S. Robinson, Anna Tai, Kyle Springer

**Affiliations:** ^1^ School of Medicine The University of Western Australia Perth Western Australia Australia; ^2^ Department of Respiratory Medicine Sir Charles Gairdner Hospital Perth Western Australia Australia; ^3^ Institute for Respiratory Health Perth Western Australia Australia; ^4^ Early Digital/Drug Intervention for COVID‐19 Treatment (EDICT), Institute for Respiratory Health, University of Western Australia Perth Western Australia Australia; ^5^ Perth USAsia Centre The University of Western Australia Perth Western Australia Australia

**Keywords:** artificial intelligence, COVID‐19, drugs, vaccination

The COVID‐19 pandemic has resulted in over 5 million deaths with an economic cost to the world estimated by the International Monetary Fund to be 28 trillion dollars.^1^ A number of COVID‐19 vaccines have been developed which, contrary to much international misinformation, have proven to be some of the most effective and safest vaccines ever developed.^2^ Despite this, it would be unwise to rely upon these vaccines alone and it remains crucial to continue to focus on developing drugs that can be taken in the earliest stages of COVID‐19 to halt progression.

Why should we not simply rely upon these vaccines?

First, the SARS‐CoV‐2 has continued to mutate and may become resistant to current vaccines. Rapid emergence and global dissemination of new virulent viral variants, such as the highly infectious ‘Delta’ variant and the B1.1.529, ‘Omicron’ variant, have highlighted the need for rapid drug and vaccine discovery. The Omicron variant harbours a large number of mutations in the spike protein compared to the Delta variant which might confer greater transmissibility, although its level of vaccine resistance is uncertain.^3^ Although the actual level of threat remains to be determined, it illustrates potential future challenges if a new variant‐specific vaccine is required to deal with such strains.^4^

A significant percentage of any population will never accept COVID‐19 vaccines and will remain vulnerable to severe COVID‐19 complications. Drugs which control COVID‐19 progression would be useful in these situations.

Second, it is considered by the WHO to be inevitable that similar viral pandemics will occur in the future.^5^, ^6^ They may be mild or may be severe such as avian flu. A key to future‐proofing us from a repeat of the COVID‐19 lockdown/mortality experience is to have drugs available which can restrain the disease, especially in the lung, turning the disease into a ‘mild flu’. If such therapies could be quickly mobilized to limit the severity of new outbreaks, it would reduce the need for lockdowns and ease healthcare burdens.

Third, it is less wealthy nations that suffer most from such pandemics due to their population numbers and density, the impact of lockdowns on subsistence living, limitations on government economic support and less access to high level health care, including intensive care unit beds and expensive drugs. The official death tolls in India and Indonesia for example, at the time of writing, are over 477,000 and 144,000 thousand, respectively. The socio‐economic impact of the pandemic in these countries has been severe. In India and Indonesia, millions of people have been pushed back into poverty. In addition, failure to control COVID‐19 in these poorer countries provides an incubator for potential virus variants. Such nations would be vastly helped by the availability of cheap, effective, rapidly deployable drugs that can restrain the disease.

The race to develop therapies for COVID‐19 has mostly focused on anti‐viral agents, two of the most promising being molnupiravir (Merck), which disrupts the replication of the virus, and paxlovid (Pfizer), which blocks viral replication by binding to an essential protease and has shown efficacy in reducing hospitalizations. The FDA has approved the use of monoclonal antibody therapy (e.g., sotrovimab, bamlanivimab, casirivimab and imdevimab) in ‘mild’ COVID‐19 patients with high‐risk features, but these therapies are often costly and require medical supervision. As such, they are not readily deployable at a population level during the emergence and transmission of different SARS‐CoV‐19 strains. Oral steroids are most effective in advanced, not early, disease.

Any new drugs will remain in limited supply, be expensive and hard to deploy around the world, even if they are listed on the WHO Essential Medicines List.

An alternative new and exciting strategy involves the use of advanced artificial intelligence (AI) to analyse the huge amount of data being gathered about COVID‐19 to identify those existing drugs which are able to target specific biological events that cause disease progression.^7^ The most advanced forms of AI‐driven drug discovery for viral diseases involve the generation of enormous data sets from diverse modalities related to the virus in question, combined with deep computational analysis to identify those disease targets that may be switched off by drugs that would not otherwise have been considered for these diseases (‘drug repurposing’). This removes the reliance on known disease pathways and opens up many more possibilities for drug discovery. Excitingly, this includes the ‘repurposing’ of sets of existing drugs which have known side‐effect profiles, are relatively cheap, are already readily available and thus rapidly deployable.

An early example is the use of AI‐based studies of COVID‐19 by a UK group which predicted that baricitinib, a drug normally used for rheumatoid arthritis, could impact on a number of components of the COVID‐19 process (Figure 1) and thus slow the progression of COVID‐19. Recent trials have confirmed that drug to be clinically effective.^8^ Such exciting developments provide hope that this approach will be applicable in the future and discover disease‐restraining drugs, which be able to be rapidly mobilized to control the outbreak and avoid lockdowns.

**FIGURE 1:**
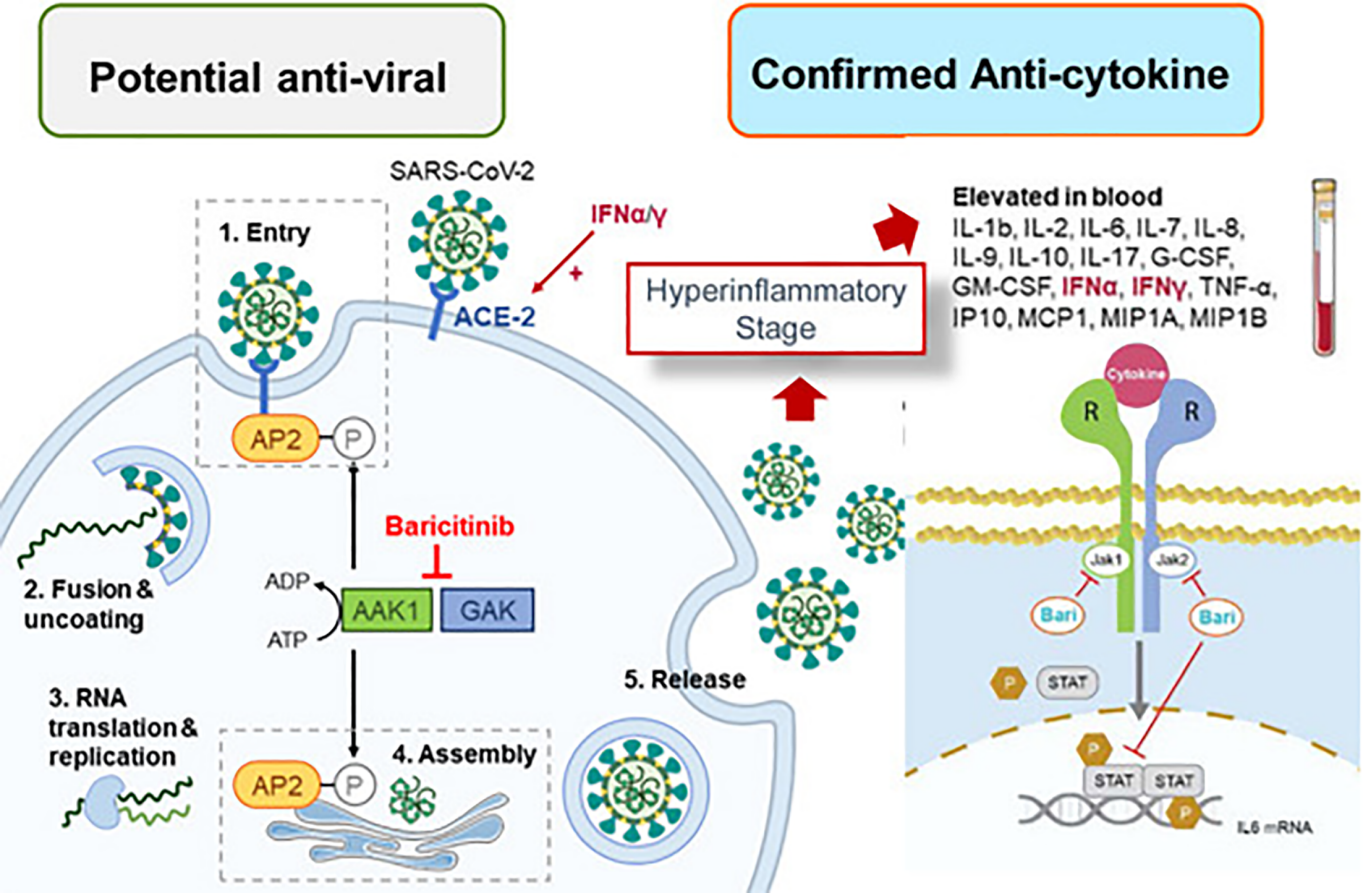
Summary of the biological effects of baricitinib underlying its artificial intelligence‐predicted role in limiting the viral replication and cytokine effects of SARS‐CoV‐2 (reproduced from Stebbing et al.^7^)

Clinical trials on early therapies to modify disease progression of COVID‐19 will require early intervention when the patient remains infectious. Undertaking clinical trials in these patients, who are in strict home isolation, requires contactless remote clinical trial platforms with robust digital monitoring. This involves deploying technologies which facilitate contactless clinical assessment (e.g., videoconferencing, eConsents), symptom tracking (eDiarys) and remote vital sign monitoring using digital monitoring devices. Whilst many of these technologies are rapidly being validated and adopted, other remote monitoring tools, particularly in biosampling (blood, saliva, throat swab, sputum), are currently in development.

In conclusion, it is important that we do not rely solely upon COVID‐19 vaccines but continue to search for novel drugs that can restrain the progression of COVID‐19 and similar diseases, especially using AI.

## CONFLICT OF INTEREST

None declared.
